# Pharmacogenetics in Psychiatry: Perceived Value and Opinions in a Chilean Sample of Practitioners

**DOI:** 10.3389/fphar.2021.657985

**Published:** 2021-04-15

**Authors:** Juan Undurraga, Ignacio Bórquez-Infante, Nicolás A. Crossley, Miguel L. Prieto, Gabriela M. Repetto

**Affiliations:** ^1^ Department of Neurology and Psychiatry, Clínica Alemana Universidad Del Desarrollo, Santiago, Chile; ^2^ Early Intervention Program, Instituto Psiquiátrico Dr J. Horwitz Barak, Santiago, Chile; ^3^ Institute of Sociology, Pontificia Universidad Católica de Chile, Santiago, Chile; ^4^ Department of Psychiatry, Pontificia Universidad Católica de Chile, Santiago, Chile; ^5^ Biomedical Imaging Center, Pontificia Universidad Católica de Chile, Santiago, Chile; ^6^ Department of Psychosis Studies, Institute of Psychiatry, Psychology and Neuroscience, King’s College London, London, United Kingdom; ^7^ Department of Psychiatry, Faculty of Medicine, Universidad de Los Andes, Santiago, Chile; ^8^ Mental Health Service, Clínica Universidad de Los Andes, Santiago, Chile; ^9^ Center for Biomedical Research and Innovation, Universidad de Los Andes, Santiago, Chile; ^10^ Department of Psychiatry and Psychology, Mayo Clinic College of Medicine, Rochester, MN, United States; ^11^ Center for Genetics and Genomics, Instituto de Ciencias e Innovación en Medicina, Facultad de Medicina, Clínica Alemana Universidad Del Desarrollo, Santiago, Chile; ^12^ Department of Pediatrics, Clínica Alemana, Santiago, Chile

**Keywords:** pharmacogenetics, psychiatry, implementation, Chile, Latin America

## Abstract

Use of pharmacogenetics (PGx) testing to guide clinical decisions is growing in developed countries. Published guidelines for gene–drug pair analysis are available for prescriptions in psychiatry, but information on their utilization, barriers, and health outcomes in Latin America is limited. As a result, this work aimed at exploring current use, opinions, and perceived obstacles on PGx testing among psychiatrists in Chile, via an online, anonymous survey. Among 123 respondents (5.9% of registered psychiatrists in the country), 16.3% reported ever requesting a PGx test. The vast majority (95%) of tests were ordered by clinicians practicing in the Metropolitan Region of Santiago. Having more than 20 years in practice was positively associated with prior use of PGx (p 0.02, OR 3.74 (1.19–11.80)), while working in the public health system was negatively associated (OR 0.30 (0.10–0.83)). Perceived barriers to local implementation included insufficient evidence of clinical utility, limited clinicians’ knowledge on PGx and on test availability, and health systems’ issues, such as costs and reimbursement. Despite the recognition of these barriers, 80% of respondents asserted that it is likely that they will incorporate PGx tests in their practice in the next five years. Given these results, we propose next steps to facilitate implementation such as further research in health outcomes and clinical utility of known and novel clinically actionable variants, growth in local sequencing capabilities, education of clinicians, incorporation of clinical decision support tools, and economic evaluations, all in local context.

## Introduction

Use of pharmacogenetics (PGx) tests to guide clinical decisions and care is appealing. From a broad perspective, this approach is intended to predict drug efficacy and adverse effects with the goal of improving clinical outcomes (Roden et al., 2019).

Genotype-guided prescribing or PGx testing considers interindividual genetic or genomic variation to inform about the likelihood of response to a specific treatment and/or of presenting adverse drug effects. It identifies genetic or genomic variants influencing drug effects, commonly via the identification of common and rare variations in genes related to pharmacokinetics or pharmacodynamics (reviewed in (Relling and Evans, 2015)). Allelic variation in genes encoding cytochrome P450 (CYP) monooxygenase system enzymes *CYP2D6* and *CYP2C19* plays an important role in commonly used psychiatric drugs (Zanger and Schwab, 2013). Also, variants in *HLA* alleles (HLA-B*15:02 and HLA-A*31:01), more commonly occurring in people of Asian ancestry, increase the risk of severe adverse reactions such as Stevens–Johnson or toxic epidermal necrolysis syndromes when using carbamazepine or oxcarbazepine (Phillips et al., 2018).

Deciding to use PGx in clinical care depends on many factors, including analytic validity, clinical validity, and clinical utility (Relling and Evans, 2015). The latter refers to whether using the test leads to improved health outcomes. In psychiatry, there is controversy regarding the clinical validity of pharmacogenetics testing for most gene-psychotropic drug pairs. Comprehensive resources such as the Pharmacogenomic Knowledge Base (PharmGKB) (PharmGKB, 2000), the Clinical Pharmacogenetics Implementation Consortium (CPIC) (CPIC, 2021), and the Dutch Pharmacogenetics Working Group (Swen et al., 2011; DPWG, 2019) aid in the implementation of PGx in clinical practice. Today, about 15% of United States FDA-approved medications contain PGx information in their label, with 38 drugs in psychiatry (Superintendencia De Salud, 2020). They include commonly used antidepressants (selective serotonin reuptake inhibitors, tricyclic antidepressants, venlafaxine, and vortioxetine), antipsychotics (aripiprazole, brexpiprazole, clozapine, haloperidol, iloperidone, risperidone, and zuclopenthixol), and mood stabilizers (carbamazepine and oxcarbazepine), among others (ISPG, 2020).

Some authors consider the available evidence insufficient to support clinical use. PGx testing is not considered, in general, as a standard of care in the field of mental health, since psychiatric disorders, such as major depressive disorder, are “determined by a large number of genes, and, except in rare cases, no single gene or limited gene set, even those for drug metabolism and drug targets, determines more than a few percent of the risk of illness or course of treatment,” with environmental factors and comorbid states and medications are important determinants (Zubenko et al., 2018). Acknowledging this, the International Society for Psychiatric Genetics (ISPG) recommended in 2019 that PGx “should be viewed as a decision-support tool to assist in thoughtful implementation of good clinical care,” and that evidence was still inconclusive to support widespread use (ISPG, 2020), but anticipated changes in the field. A more recent (2021) consensus by experts convened by the ISPG considers that current evidence does support the use of PGx testing for cytochrome P450 genes CYP2D6 and CYP2C19 to inform selection and dosing of commonly used antidepressant and antipsychotic medication, as well as testing for HLA-A and HLA-B when using carbamazepine, HLA-B for oxcarbazepine, PYP2C9, and HLA-B for phenytoin, and when a mitochondrial disorder or a urea cycle disorder is suspected, POLG, OTC, and CSP1 for valproate use (Bousman et al., 2021). Consequently, the authors predict that PGx testing will become a relevant tool in psychiatry.

In addition to information on validity and utility, other factors also impact implementation, such as physician awareness, availability and accessibility of testing, associated costs and reimbursement, and cost-effectiveness analyses in local contexts among others (Relling and Evans, 2015; Luzum et al., 2017). Some studies have assessed physicians’ attitudes toward PGx. A recent survey on primary care and mental health providers enrolled in the Veterans Affairs’ PRecision Medicine In MEntal Health Care (PRIME Care) study (Hull et al., 2019), revealed that less than 15% of 342 participants had ordered a PGx test to guide psychotropic medication in the previous year. Earlier also performed in the United States showed similar results: 12.9% of more than 10,000 responders of a nationwide survey of physicians in different specialties (Stanek et al., 2012) and 20% of 597 United States internists and family medicine practitioners (Haga et al., 2012), although these studies did not describe the specific area of ordered tests. Other studies show that a substantial proportion of psychiatrists consider that PGx testing will become a useful aid in drug prescribing (Thompson et al., 2015; Walden et al., 2015).

Most work on discovery, applications, and implementation of PGx has been carried out in developed countries, and while the global information is based on larger samples than in Latin America, there is limited information on use and views among practitioners in this region. For this reason, we conducted a survey on opinions about current practices, perceived value and barriers to clinical use of PGx testing, in a sample of clinical psychiatrists from Chile.

## Methods

### Sample and Survey

We conducted an anonymous, cross-sectional survey via e-mail among clinical psychiatrists working in Chile. Contact information was obtained from directories of medical societies and professional associations, and smaller databases from the authors. The survey was approved by Research Ethics Committee at Facultad de Medicina Clinica Alemana Universidad del Desarrollo, and participants gave informed consent.

The survey was sent in November and December 2020, with an explanatory cover letter, an informed consent form, and a 10-question survey exploring opinions and barriers toward use of PGx in their clinical practice and future intention to use. Prior to submission, the questions were reviewed by a psychiatrist and a nonpsychiatrist physician, for comprehension and readability. The survey was sent in Spanish. The translated questions can be found in Supplementary Table S1.

### Statistical Analysis

A descriptive analysis was performed to report the participant characteristics and their responses, using frequencies and percentages. Association analysis was made using the chi-square test for categorical variables. The included variables predicting usage of PGx were 1) practice in the Metropolitan Region of Santiago; 2) practice in the public system; 3) practice at a university or research center; 4) practice at private medical center; 5) practice in adult psychiatry; and 6) years in practice. All analyses were conducted using Stata v16 (StataCorp. 2019, Stata Statistical Software: release 16. College Station, TX: StataCorp LLC).

## Results

At the time of the survey submission, there were 2093 registered psychiatrists in Chile, 1,022 of them (49%) in the Metropolitan Region of Santiago (FDA, 2021). The survey was sent to 546 psychiatrists registered in medical societies and professional associations. Responses were received from 123 (5.9% of registered psychiatrists and 22.5% response rate). Answers for some questions were not mutually exclusive; for example, a psychiatrist could have more than one practice setting or see patients of different age groups. Demographic characteristics are described in Table 1. Briefly, ninety-two participants (74.8%) worked in Santiago and the remainder in nine of the other fifteen regions of the country. The majority worked in combinations of public, private, and academic health settings, and 84% were adult psychiatrists. Close to half of respondents, 45.5%, had less than ten years in practice, and 61% had participated in research in the previous five years.

**TABLE 1:** Sample characteristics and associations with prior use of PGx tests.

	Total		Ever used pharmacogenetic tests				p-value
			No N = 103 (83.7%)		Yes N = 20 (16.3%)		
	N	%	N	%	N	%	
Metropolitan region**	92	74.8	79	70.9	19	95.0	0.02
Practice at (% yes)^a^							
Public system**	67	54.5	61	59.2	6	30	0.02
University or research center	27	22	21	20.4	6	30	0.34
Private medical center*	90	73.2	72	69.9	18	90	0.06
Nonprofit foundation	5	4.1	4	3.9	1	5	0.81
In training	3	2.4	3	2.9	0	0	0.44
Other	16	13	13	12.6	3	15	0.77
Clinical practice (% yes)^a^							
Child psychiatry	19	15.4	15	14.6	4	20	0.53
Adolescent psychiatry	31	25.2	25	24.3	6	30	0.59
Adult psychiatry	104	84.6	87	84.5	17	85	0.95
Years in practice**							
0 to 10	56	45.5	51	49.5	5	25	
11 to 20	26	21.1	22	21.4	4	20	
21 to 30	22	17.9	19	18.4	3	15	0.02
31 to 40	16	13	9	8.7	7	35	
41 or more	3	2.4	2	1.9	1	5	
Participated in research in past 5 years							
Yes	75	61	62	60.2	13	65	0.68

Note: N = 123. ***p < 0.01, **p < 0.05, *p < 0.1.

^a^ More than one answer permitted.

Twenty participants (16.3%) had ever requested PGx tests for psychotropic medication prescribing, most of them (80%), more than once. The median number of times they had requested a test was 3, with a range of 1–10. Most of the requests (90%) were made by physicians who declared working in the private healthcare system. The reported reasons for ordering a PGx test were to predict drug response (85%) or side effects (40%), or to explain observed side effects (25%). No tests by these psychiatrists were ordered per patient’s request or other reasons not specified in choices in the survey.

Factors significantly associated with prior use of PGx testing included working in the Metropolitan Region (p 0.02, OR 7.81, 95% CI 1.00–60.98) and having 21 or more years in practice (vs. 10 or less years) (p 0.02, OR 3.74, 95% CI 1.19–11.80). In fact, only one clinician who reported use of PGx testing did not work in the Metropolitan Region. Practicing in the public health system was negatively associated with PGx testing (p 0.02 OR 0.3 [95% CI, 0.10–0.83]).

The main reason reported for not having ordered PGx testing was lack of awareness of tests that could be relevant for one’s own clinical practice (62.1%), followed by costs (44.7%) and by perception of lack of utility (23.3%).

Perceived barriers to use of PGx included lack of personal knowledge about pharmacogenetics (51.2%), high cost of available exams (48.8%), and lack of availability of pharmacogenetic tests in Chile (46.3%). We grouped surveyed barriers in three main areas: 1) available evidence, 2) provider-related barriers, and 3) system-related barriers. Provider-related barriers were more common among those who have not used PGx (88.4 vs. 65.0% in past users, p < 0.01), whereas healthcare system barriers were mentioned more frequently by those that have used tests in the past (90.0 vs. 78.6% in not users), although this difference was not statistically significant (*p* = 0.24) (Table 2). Perception of lack of evidence of clinical utility was mentioned more frequently by prior users (55%) than nonusers (25.2%) (*p* < 0.01). Despite recognition of these gaps, the majority (80%) considered likely that they will use PGx tests in their practice in the next 5 years, independent of having used them or not in the past (p 0.81).

**TABLE 2:** Perceived barriers to pharmacogenetic tests use.

	Ever used pharmacogenetic tests				Total		p-value
	No N = 103 83.7%		Yes N = 20 16.3%				
	N	%	N	%	N	%	
What are the main barriers to the use of pharmacogenetic tests? (% yes)							
Provider-related barriers ***	91	88.4	13	65	104	84.6	0.00
Lack of personal knowledge about pharmacogenetics	56	54.4	7	35	63	51.2	0.11
Not knowing where they are available **	51	49.5	4	20	55	44.7	0.02
Not knowing how to request them **	38	36.9	2	10	40	32.5	0.02
Complexity to request tests	13	12.6	2	10	15	12.2	0.74
Complexity to interpret the results **	7	6.8	5	25	12	9.8	0.01
Healthcare system-related barriers	81	78.6	18	90	99	80.5	0.24
High cost of available tests **	46	44.7	14	70	60	48.8	0.04
Lack of availability of pharmacogenetic tests in Chile	49	47.6	8	40	57	46.3	0.53
Lack of coverage by health systems	41	39.8	11	55	52	42.3	0.2
Lack of evidence of clinical utility ***	26	25.2	11	55	37	30.1	0.00
Other	5	4.9	0	0	5	4.1	0.31
Will incorporate the use of pharmacogenetic tests in its clinical practice in the next 5 years							
Yes	73	80.2	14	77.8	87	79.8	0.81

*** *p* < 0.01, ** *p* < 0.05, * *p* < 0.1.

## Discussion

This work represents the first survey of clinicians in Chile regarding the use and perceived barriers of PGx testing in psychiatric practice. Most respondents worked as adult psychiatrists in the Metropolitan Region of Santiago. However, the full sample included a broad spectrum of geographic locations, practice settings, and years of clinical experience. Prior use of PGx testing was reported by 16%, similar in proportion to studies done in the United States (Haga et al., 2012; Stanek et al., 2012; Hull et al., 2019) but each clinician in our survey had ordered tests 10 times or less, evidencing that, overall, its use was quite limited.

Factors associated with utilization of PGx testing included practice in the Metropolitan Region of Santiago and in private healthcare settings. These results could be related to the fact that tests are not currently available in certified clinical laboratories in Chile; therefore, samples need to be sent to laboratories abroad. This involves logistical challenges to order and to arrange for shipment and payments, processes that must be organized by the physician since these centers do not have local offices or contracts. In addition, testing performed out of the country is not covered by private or public insurance and it poses a significant out-of-pocket expense for patients (Villalobos Dintrans, 2018). Also, given the added shipment days, turnaround times for results are long, limiting the ability to guide prescription decisions, especially in acute care settings. Finally, and although not explored in this study, the lack of standards for PGx panels results in differences in the available ones; this heterogeneity may also add to challenges in ordering decisions (Fan and Bousman, 2020; Bousman et al., 2021).

Being in mid to late career stages (more than 20 years in practice) was positively associated with prior use, which suggests that knowledge in the field may have been acquired through continuing education or exposure to international research. PGx is not formally included the curriculum in medical schools or residency training programs in Chile. The reasons reported to use tests were mostly reactive to specific prescribing situations, that is, prediction of effects or explanation of side effects of particular drugs. No mention was made of preemptive testing; however, this was not explicitly explored in this study.

Among barriers to PGx testing was the perception of insufficient evidence of clinical utility. This was mentioned more frequently by prior users than nonusers, who may be more aware of the current limitations. As stated in the introduction, the clinical value of PGx testing in psychiatry is still a matter of debate and an evolving issue. The recently published review and updated consensus on PGx testing in psychiatry by a group of international experts assembled by the ISPG might help change this perception and advance in expanding the use of PGx testing in clinical settings for specific validates genes and gene-pair drugs (Bousman et al., 2021).

Most participants predicted that they will include PGx testing in their clinical practice in the next five years. This finding mirrors opinions of psychiatrists in the United States and Canada (Thompson et al., 2015; Walden et al., 2015) where more than 80% of respondents in these studies consider that PGx will become a common standard in practice. Similar favorable opinions toward PGx testing were reported in a study of physicians in Jordan (Muflih, 2017). Nevertheless, the respondents of our study also recognized the presence of barriers to clinical use. In addition to insufficient evidence for clinical utility, other perceived difficulties were related to clinicians’personal knowledge about PGx, the logistics involved in requesting tests, and health system barriers, such as cost and reimbursement. These are also similar to the barriers identified by the largest published nationwide survey in the United States (Stanek et al., 2012). Another important issue is that, although CPIC and FDA guidelines are available for specific drug pairs, knowledge of their application and results in terms of health outcomes in Latin-American populations is practically nonexistent. In addition, there are differences in allelic composition and frequencies in several relevant genes, compared to European and other populations (Naranjo et al., 2018; Rodrigues-Soares et al., 2020), which may have an impact on clinical use in the region.

To address these limitations, several next steps are needed. Although most of the described challenges are similar to those described by practitioners in North America, there is a need to generate local information. Research on validation of known variant drug pairs in Latin-American populations and discovery of potential novel actionable associations are crucial; since due to the admixed genetic nature of local populations, findings from participants with other ancestries cannot be immediately transferred to the region. For instance, the RIBEF-CEIBA Network Consortium published a study analyzing CYP2D6, CYP2C9, and CYP2C19 genetic polymorphisms on 6,060 healthy individuals from Ibero-America, classified according to their self-reported ancestry, and found that Amerindians, that contribute to the admixed populations in the region, had significant differences in allelic frequencies with other ethnic groups, and also between native populations from the north and the south (Naranjo et al., 2018). These differences show the need for further local research in clinical applications and outcomes.

Once validity and utility are established in local context and training of clinicians is necessary. This requires the incorporation of PGx content in curricula in medical schools, graduate, and continuing medical education since this field is rapidly changing. Ease of access to pertinent tests is another relevant factor, and efforts are needed to increase local sequencing and interpretation capacities for accurate and rapid turnaround genotyping. Development and inclusion of clinical decision support tools that also consider nongenetic information, and their integration into health records are also facilitators, particularly, since as mentioned above, prediction of beneficial and side effects is of multifactorial nature. Economic analysis is also needed to inform coverage and reimbursement decisions for health services. Differences in allelic frequencies across global populations can impact on cost-effectiveness predictions in different countries (Zhou et al., 2021).

Limitations of this study include that it is a convenience sample, with a relatively small sample size and low response rate, representing only 6% of psychiatrists working in Chile. Nevertheless, the number of respondents were similar to previous similar surveys in more populated countries such the United States and Canada (Thompson et al., 2015; Walden et al., 2015), and to a study of physicians’ response rate to web-based surveys, in the range of 27–46%, with the lower figure for psychiatrists (Cunningham et al., 2015). Most psychiatrists were affiliated both to private and public sectors and this study did not explore in which specific setting the tests were requested. It is possible that responses were mostly from physicians that may be more aware of PGx and therefore more inclined to participate. These results limit generalizability of the conclusions of the study. In addition, the survey did not explore whether the results of PGx testing had modified therapeutic decisions and/or patient outcomes in those cases in which it had been used.

In summary, this exploratory study shows that the current uptake of PGx testing by psychiatrists in Chile is low, but there is interest in the area and in potential incorporation in clinical practice. To bridge the gap between current and future appropriate use, local research and implementation strategies are needed. Understanding the scope and problems of PGx implementation can also benefit from collaboration throughout Latin America since the similar challenges are shared by other countries in the region (Abou Diwan et al., 2019).

## Data Availability

The raw data supporting the conclusions of this article will be made available by the authors, without undue reservation.
