# Supplementary material for: Pharmacogenetics in Psychiatry: Perceived Value and Opinions in a Chilean Sample of Practitioners
Source: Front Pharmacol. 2021 Apr 15;12:657985. doi: 10.3389/fphar.2021.657985 (PMC8082421; doi:10.3389/fphar.2021.657985)
Supplement: Supplementary file 1 [file datasheet1.docx]

Supplementary Material

# Supplementary Data

Supplementary Table 1. Survey (translated questions)

SURVEY OF OPINIONS ON THE USE OF PHARMACOGENETICS IN PSYCHIATRY

1. What region do you work in? (optional)

2. In which setting do you see patients? (check all that apply)

a. Public health system

b. Academic or Research Center

c. Private Medical Center

d. Private individual practice

e. Foundation

f. Other. If so, which one?

3. What is the predominant age group of patients you care for? (check all that apply)

a. Children

b. Adolescents

c. Adults

4. How many years have you been in clinical practice?

a. 0-10 years

b. 11-20 years

c. 21-30 years

d. 31-40 years

e. More than 40 years

5. Have you participated in research in the last 5 years?

a. Yes

b. No

6. Have you requested a pharmacogenetics test for a patient?

a. Yes

b. No

7. If yes:

a. For what purpose have you requested it?

i. To predict favorable response to a certain medication

ii. To predict the onset of side effects

iii. To explain adverse effects

iv.At the request of the patient

v. Other reason… Which one?

b. How many times have you requested pharmacogenetic tests?

8. If your answer is "no":

a. Why haven't you requested pharmacogenetic tests?

i. Due to cost

ii. I do not consider them useful for my clinical practice

iii. I am not familiar with tests specific to my clinical practice

iv. Other reason… Which one?

9. In your opinion, what are the main barriers to the use of pharmacogenetic tests? Check the 3 you consider most important:

a. Lack of evidence of clinical utility

b. Lack of personal knowledge about pharmacogenetics

c. Unavailability of pharmacogenetic tests in Chile

d. Lack of coverage by health systems

e. High cost of available tests

f. Complexity in requesting tests

g. Complexity in interpreting the results

h. Other….Which?

10. Do you think that over the next 5 years you will incorporate the use of pharmacogenetic tests into your clinical practice? Yes/no
